# The Effects of the COVID-19 Pandemic Lockdown on Eating, Body Image, and Social Media Habits Among Women With and Without Symptoms of Orthorexia Nervosa

**DOI:** 10.3389/fpsyg.2021.716998

**Published:** 2021-12-15

**Authors:** Keisha C. Gobin, Jennifer S. Mills, Sarah E. McComb

**Affiliations:** Department of Psychology, York University, Toronto, ON, Canada

**Keywords:** COVID-19, orthorexia nervosa, eating, social media, body image

## Abstract

The COVID-19 pandemic is negatively impacting people’s mental health worldwide. The current study examined the effects of COVID-19 lockdown on adult women’s eating, body image, and social media habits. Furthermore, we compared individuals with and without signs of orthorexia nervosa, a proposed eating disorder. Participants were 143 women, aged 17–73 years (*M* = 25.85, *SD* = 8.12), recruited during a COVID-19 lockdown in Canada from May-June 2020. Participants completed self-report questionnaires on their eating, body image, and social media habits during the pandemic. The Eating Habits Questionnaire (EHQ) assessed symptoms of orthorexia nervosa. Compared to the period prior to lockdown, women with higher total orthorexia nervosa scores reported eating a lot more than usual, feeling greater pressure to diet and lose weight, thinking about food more often than usual, experiencing greater weight gain, and perceiving more pressure from social media specifically to lose weight and to exercise, compared to their healthy counterparts. We examined associations between individual EHQ subscales and perceived changes to eating and weight. Women who scored high on EHQ-Problems reported seeing more weight loss content on their social media than those who reported fewer orthorexia nervosa symptoms. Conversely, those who scored low on EHQ-Feelings reported feeling a lot less pressure to lose weight, somewhat less or a lot less pressure to lose weight or to exercise from social media specifically, and trended toward less laxative use during lockdown, compared to those who scored higher on orthorexia nervosa. And those who scored low on EHQ-Knowledge reported feeling somewhat less or a lot less pressure to lose weight than those who reported more orthorexia nervosa symptoms. Together, the findings suggest that women with symptoms of orthorexia nervosa are experiencing an exacerbation of disordered eating thoughts and behaviors during COVID-19, and that social media may be a contributing factor.

## Introduction

The ongoing COVID-19 pandemic has negatively impacted people’s health worldwide. In addition to the toll the disease is having on people’s physical health, lockdowns and social distancing restrictions aimed at curbing the spread of the virus are associated with adverse mental health outcomes. A meta-analysis by Salari et al. (2020) of 14 studies from nine different countries concluded that COVID-19 has increased the prevalence of mental health disorders globally. The COVID-19 outbreak in China in early 2020 led to an increase in anxiety, depression, self-harm, suicide attempts, especially among younger people (Qiu et al., 2020), and post-traumatic stress (Liu et al., 2020). The number of days a person spent in lockdown, proximity of positive cases, and having to relocate were associated with higher levels of psychological distress in the early part of the pandemic in Italy, including higher post-traumatic stress symptoms (Di Giuseppe et al., 2020). Some individuals in the general population are more at risk of experiencing negative mental health effects of the pandemic. A cross-cultural study of 2,787 adults by Prout et al. (2020) found that in addition to younger age, somatization tendencies and less reliance on adaptive defense mechanisms were associated with greater levels of distress during the pandemic. Novotný et al. (2020) found that a COVID-19 lockdown produced a surge in mental distress across age groups, but that increases in distress were more severe in women. The same study found that illness perception, feelings of loneliness, low levels of resilience and resilient coping, and several lifestyle components were associated with a significant increase in mental distress during a lockdown. Another study in the early months of the pandemic found that lower levels of mindfulness were positively correlated with higher levels of overall psychological distress (Conversano et al., 2020).

### COVID-19 and Diet-Related Lifestyle Changes

In addition to mental health, people’s diet-related lifestyle behaviors have also been impacted by the pandemic. In many places in the world, there have been a series of lockdowns that include the closure of non-essential workplaces, schools, gyms, and dining. Accordingly, people are spending far more time than usual working, exercising, and eating at home. A study of Worldwide search trends on the internet revealed that during the pandemic people are searching for more information regarding food delivery, take away restaurant services, and nutritional supplements (Mayasari et al., 2020). A large study of adults in France during lockdown in April-May 2020 found that most respondents reported decreased physical activity, increased sedentary time, increased snacking, decreased consumption of fresh food, and increased consumption of sweets, cookies, and cakes during that period (Tanguy et al., 2021). At the same time, a sizeable proportion of respondents reported increased home cooking and increased physical activity (Tanguy et al., 2021). Other studies have also found that healthy eating increased during stay-at-home restrictions and time spent in physical activity decreased (Chopra et al., 2020; Flanagan et al., 2020). A study from Netherlands (Poelman et al., 2021) found that changes to eating behaviors and food purchases were moderated by age and body mass index (BMI). Younger, but not older, participants were likely to report changes to their eating during lockdown, with an equivalent number of younger participants saying that they were eating healthier vs. less healthy foods than usual. In the same study, individuals with obesity reported purchasing and eating more chips/snacks than usual during lockdown, as well as experiencing more anxiety and weight gain (Poelman et al., 2021).

There have also been notable changes in perceived weight changes during this pandemic, possibly attributed to increased food consumption. In April 2020, during lockdowns across Italy, a survey of individuals aged 12–86 years revealed that about half of respondents perceived having gained weight during lockdown (Renzo et al., 2020). Another study in Italy (Scarmozzino and Visioli, 2020) revealed that 52.9% of respondents reported an increase in their total food consumption during the duration of confinement. Specifically, there was an increase in self-reported consumption of “comfort food”: 23.5% of individuals reported eating more salty snacks during lockdown, while 42.5% of individuals reported an increase in the amount of ice-cream, chocolate, or desserts consumed under lockdown. Approximately 20% of respondent reported an increase in their weight during lockdown (Scarmozzino and Visioli, 2020). A Chilean study of adults from the general population conducted during that country’s month of strict lockdown to the start of the pandemic found that approximately half of adult women reported that their weight had changed during lockdown; more of them thought they had gained weight (38%) than lost weight (14%) (Reyes-Olavarría et al., 2020). Research from Poland during COVID-19 found that 43% of participants reported eating more meals than normal, 52% reported increased snacking, 30% reported weight gain, and 18% reported weight loss (Sidor and Rzymski, 2020). An American study of men and women similarly found mixed results in terms of weight change during the pandemic, but that weight gain was predicted by eating when bored (Zachary et al., 2020). In addition to boredom, there are several possible reasons for increased food consumption during the pandemic. Working or studying from home may lead to an absence of routines and disrupt normalized eating schedules, which, in turn, can increase snacking or binging (Todisco and Donini, 2020). And government recommendations to limit the frequency of grocery shopping and the perceived scarcity of certain food products may increase a focus on food and “stocking up,” which could increase the frequency of binge eating (Waters et al., 2001; Rodgers et al., 2020).

As reviewed above, there is evidence that people’s eating habits changed during the early part of the pandemic. Social media content reflects people’s concern about possible weight gain due to diet-related lifestyle changes. Since the COVID-19 pandemic started, there has been a documented increase in “fatphobic” content on social media, such as hashtags referencing the “Quarantine 15” weight gain, suggesting widespread and unwanted weight gain (Pearl, 2020). Stigmatizing posts may cause increases in shape and weight concerns, dieting, excessive exercising, or purging behaviors among vulnerable individuals. Research has shown that there was a tremendous increase in social media use in the first months of the pandemic (Kemp, 2020), which has previously been linked to negative body image and dieting behavior (Hogue and Mills, 2019).

### COVID-19 and Eating Disorders

It has been theorized that individuals with eating disorders (EDs) may be especially susceptible to greater levels of distress due to pandemic restrictions and an exacerbation of pre-existing ED symptoms prior to COVID-19 (Touyz et al., 2020). Changes to routine, reduced social contact and access to professional support/treatment for their disorder, along with food scarcity, may serve to worsen or trigger ED symptoms (Sheng et al., 2021). In an Australian study, Phillipou et al. (2020) recruited 180 participants who self-identified as having an eating disorder of any kind, as well as individuals from the general population. Results among individuals with an ED showed that 67% reported having restricted food intake more since the start of the pandemic, while 9% reported having restricted food intake less than prior to the pandemic. Furthermore, 21% reported binging more during COVID, while 10% reported less binging during these times. And 18% reported purging more during COVID, while 5% reported purging less than before the pandemic. In terms of physical activity, 49% of individuals with an eating disorder reported having exercised more during the pandemic, while 33% of individuals reported having exercised less. Among respondents in that study from the general population, increased restricting and binge eating were also reported; however, individuals without an eating disorder reported less exercise relative to before the pandemic (Phillipou et al., 2020). Other research has similarly shown that individuals with binge eating report a worsening of symptoms during COVID-19 (Fernández-Aranda et al., 2020; Schlegl et al., 2020; Termorshuizen et al., 2020).

### COVID-19 and Orthorexia Nervosa

While emerging research reveals the effects of the COVID-19 pandemic on individuals with traditional ED diagnoses (i.e., anorexia nervosa, bulimia nervosa, binge eating disorder), its effects on individuals reporting symptoms of orthorexia nervosa have yet to be examined. The term orthorexia nervosa (ON) was first coined over two decades ago by Steven Bratman, capturing people who are concerned about the “cleanliness” of their food and, as a result, restrict or completely eliminate entire categories of food (e.g., meat, dairy) or ingredients (e.g., sugar, preservatives) they perceive to be impure and/or unhealthy. They spend considerable time scrutinizing food sources for possible contamination or toxins, as well as avoiding specific nutrients, such as saturated fats, proteins, and carbohydrates (Bratman and Knight, 2000). Complex eating rituals and internalized beliefs about the dangers of combining certain foods may arise. Individuals with ON may have distorted beliefs around digestion and the need to eat in a highly prescribed way to avoid health problems. Although improved health is often the purported goal of ON, it was observed that such rigid and restrictive eating practices may lead to malnutrition, social isolation, and psychological distress (Fidan et al., 2010).

ON has come to be conceptualized as an ED (Janas-Kozik et al., 2012; Barthels et al., 2015). Bratman (2017) and others have since emphasized that healthy eating alone does not imply pathology and must be extreme to be clinically significant, and proposed criteria have been theorized (Moroze et al., 2015). Dunn and Bratman (2016) have proposed a revised set of diagnostic criteria based on a review of the literature that clarify that orthorexic tendencies are pathological when food theories intensify and escalate over time into an extreme fixation.

People with ON and anorexia nervosa share high trait perfectionism, high trait anxiety, and a high need to exert self-discipline and control (Fidan et al., 2010; Barnes and Caltabiano, 2017). In one study, the prevalence of ON symptoms was higher among individuals with ED symptoms, suggesting that ON might be either a precursor for or a residual of an eating disorder (Brytek-Matera et al., 2020). Given the associated risks for individuals reporting symptoms of ON, it is essential to understand the level of risk for this population during the current global pandemic.

### The Current Study

The current study’s main objective was to examine reported changes to adults’ eating habits, exercise, and social media use during the COVID-19 pandemic and a lockdown period in Canada during the summer of 2020. We examined only women since dieting, drive for thinness, and disordered eating are strongly influenced by gender and are disproportionately prevalent among women (Mills et al., 2018). We needed to collect an adequate sample size during the critical lockdown period of study and, thus, focused on recruiting female participants. Furthermore, this study compared changes between individuals with and without symptoms of ON. Given the overlap between ON and disordered eating, it was hypothesized that individuals high on ON symptoms would report increased disordered eating habits, worsened body image, and would be more negatively affected by social media during a COVID-19 lockdown as compared to those low on ON symptoms. Most of the research to-date on the effects of lockdowns were conducted during the hardest hit and earliest outbreaks of COVID-19. The current study took place a few months into the pandemic, after some habituation to the initial change in circumstances had presumably occurred.

## Materials and Methods

### Participants

Participants (*N* = 170) were recruited either through an undergraduate research participant pool at York University in Toronto, Canada (*n* = 85) or through online community advertisements (*n* = 58). Twenty-seven participants were excluded because of significant missing data, resulting in a final sample of 143 female participants. Participant ages ranged from 17 to 73 years (*M* = 25.85, *SD* = 8.12). The self-reported ethnic distribution of the sample was primarily Caucasian (51.7%), followed by South Asian (11.9%), Middle Eastern (9.1%), East Asian (6.3%), Asian (6.3%), Other (5.6%), Black (4.9%), and Latino/Hispanic (3.5%). Participants’ self-reported body mass index (BMI = kg/m^2^) scores ranged from 15.20 to 52.40 (*M* = 25.37, *SD* = 7.45). The majority of the sample (35.0 %) had completed some university as their highest level of education, followed by an undergraduate degree (22.4%), high school diploma (19.6%), college diploma (12.6%), master’s degree (7.0%), and doctorate degree (2.8%).

### Measures

#### Orthorexia Nervosa

The Eating Habits Questionnaire (EHQ; Gleaves et al., 2013; Oberle et al., 2017) was used to assess ON symptomology. The EHQ is a 21-item self-report questionnaire that is composed of three distinct subscales. The first subscale is composed of eight items which measure knowledge of healthy eating behaviors (EHQ-Knowledge; e.g., “*I am more informed than others about healthy eating*”; *a* = 0.75). The second subscale is composed of nine items, which measure problems due to healthy eating (EHQ-Problems; e.g., “*In the past year, friends or family members have told me I’m overly concerned with healthy eating*”; *a* = 0.91). The third subscale is composed of four items, which measure feeling positively about healthy eating (EHQ-Feelings; e.g., “*I feel in control when I eat healthily*”; *a* = 0.72). Higher scores reflect stronger endorsement of ON symptoms. Participants were asked to respond to each item on a four-point scale from *False, not at all true* to *Very true*. Internal consistency in the current study was excellent for the global score (*a* = 0.93).

#### Eating, Exercise, and Social Media Habits During Quarantine

Because of the uniqueness of the event in question, the authors created a self-report questionnaire designed to measure changes in eating, exercise, and social media habits during the pandemic. Participants were asked to indicate whether “*during the last few weeks since the COVID-19 (coronavirus) lockdown began*” they had experienced any increases or decreases in dieting and food consumption compared to usual, purging, and laxative use habits, as well as changes to exercise and social media habits compared to usual. Participants responded on a five-point scale (1–5): a lot less, somewhat less, about the same, somewhat more, and a lot more.

### Procedure

Participants were recruited during a pandemic lockdown period between May 29th to June 9th, 2020. Starting on March 17, 2020, the province of Ontario, where the study was conducted, was in a declared state of emergency due to COVID-19. Residents were advised to practice social distancing and stay home, group gatherings were limited to five people or fewer, and schools, universities, gyms, and all non-essential businesses were closed. Grocery stores and take-out food businesses were allowed to remain open. These restrictions were all still in place during data collection.

Ethics approval was granted by the York University Human Participants Review Committee. Eligible participants volunteered for the study through an online experiment management system or through advertisements on the internet. Upon signing up, participants provided informed consent online and then were asked to complete the measures listed above as well as demographic information. Participants either received partial course credit or the chance to win a gift card.

### Data Analysis

Because there are no established diagnostic criteria for ON, we categorized participants into high and low ON symptom groups based on a median split on the EHQ. This type of categorical approach to assessment allows for between-group comparisons and simpler language when describing different types of individuals, and is used in the assessment of other eating-related behaviors, such as the identification of dieters vs. non-dieters (see Polivy et al., 2020). A median split was similarly used to independently examine the EHQ subscales of knowledge about healthy eating behaviors, problems due to healthy eating, and feeling positively about healthy eating. A chi-square test of independence was conducted between the two groups on each potential change of interest. Items with at least 80% of expected frequencies greater than five were examined at all five levels of the Likert scale (Field, 2013). For items with expected frequencies less than *n* = 5, groups were collapsed to either two or three levels. For two levels, the groups were collapsed to “about the same or less” (scale items 1, 2, and 3) or “more than usual” (scale items 4 and 5). For three levels, the groups were collapsed to “less than usual” (scale items 1 and 2), “about the same” (scale item 3), and “more than usual” (scale items 4 and 5). Significant chi-square analyses were followed up with *post hoc z*-tests to determine where the significant interaction occurred. One-way analysis of variance (ANOVA) was used to compare differences between the groups on engaging in a COVID-19 diet, hours spent viewing diet-related social media accounts per day, and viewing social media content about losing weight.

## Results

### High vs. Low ON Symptom Groups

A one-way ANOVA demonstrated that there was no significant difference in age between those high and low on ON symptoms, *F* (1,142) = 0.74, *p* > 0.05. There was a significant difference in self-reported BMI, *F* (1,138) = 6.28, *p* = 0.013, whereby those high on ON symptoms had a higher BMI (*M* = 26.91, *SD* = 8.08) compared to those low on ON symptoms (*M* = 23.80, *SD* = 6.43). BMI was not adjusted for statistically for ensuing analyses (i.e., used as a covariate), so as not to obscure meaningful group differences. There were no significant differences on age or BMI for the two groups when examined for each EHQ subscale.

### Eating Attitudes and Behaviors

Significant differences were found between participants high and low on ON symptoms in regard to thoughts about food during the COVID-19 lockdown, χ^2^(4) = 16.79, *p* = 0.002, Cramer’s V = 0.35. Specifically, women high on ON symptoms reported thinking about food “a lot more” than usual compared to those low on ON symptoms (*z* = −3.71, *p* < 0.001). In other words, thinking about food “a lot more” than before lockdown was disproportionately common among women high on ON symptoms. There were also significant differences in the amount of food participants reported eating during lockdown compared to usual, χ^2^(4) = 15.89, *p* = 0.003, Cramer’s V = 0.33, such that those high on ON symptoms were more likely to report eating “a lot more” compared to those low on ON symptoms (*z* = −2.05, *p* = 0.040) and those low in ON tendencies were more likely to report eating “about the same” compared to those high in these tendencies (*z* = 3.30, *p* < 0.001). In other words, eating “a lot more food” than before lockdown was disproportionately common among women high on ON symptoms. Additionally, there were significant difference between the groups in regard to perceived weight change since the start of the COVID-19 lockdown, χ^2^(2) = 11.25, *p* = 0.004, Cramer’s V = 0.28. Those high on ON symptoms were more likely to report “somewhat to much greater” increases in weight compared to the low ON symptoms group (*z* = −2.46, *p* = 0.014), while those low on ON symptoms were more likely to report having “about the same” weight since the start of the lockdown as compared to those high in these symptoms (*z* = 3.24, *p* = 0.001). In other words, perceiving having gained weight during lockdown was disproportionately common among women high on ON symptoms. See Table 1 for statistical test results.

**TABLE 1:** Items related to eating attitudes and behaviors (chi-squared tests).

Items		χ ^2^	*df*	*z*	*p*	Cramer’s V
Thinking about food more than usual		16.79	4	–	0.002	0.35
	A lot less	–	–	1.93	0.054	–
	Somewhat less	–	–	−0.32	0.749	–
	About the same	–	–	1.82	0.069	–
	Somewhat more	–	–	0.05	0.960	–
	A lot more	–	–	−3.71	<0.001	–
Eating compared to usual		15.89	4	–	0.003	0.33
	A lot less	–	–	−1.95	0.051	–
	Somewhat less	–	–	0.29	0.662	–
	About the same	–	–	3.30	<0.001	–
	Somewhat more	–	–	−1.01	0.313	–
	A lot more	–	–	−2.05	0.040	–
COVID weight change		11.25	2	–	0.004	0.28
	A lot lower or somewhat lower	–	–	−1.19	0.230	–
	About the same	–	–	3.24	0.001	–
	Somewhat higher to much higher	–	–	−2.46	0.014	–

### Dieting Attitudes and Behaviors

Significant differences between the two ON symptom groups were found in regard to perceived pressure to lose weight during lockdown, χ^2^(4) = 27.51, *p* < 0.001, Cramer’s V = 0.46. Those high on ON symptoms were more likely to report “a lot more” perceived pressure to lose weight compared to their low ON symptom counterparts (*z* = −4.05, *p* < 0.001), while those low on ON symptoms were more likely to report “about the same” (*z* = 2.32, *p* = 0.020) or “a lot less” (*z* = 3.64, *p* < 0.001) pressure to lose weight since the lockdown began as their high ON symptom counterparts. In other words, perceiving “a lot more” pressure to lose weight during lockdown was disproportionately common among women high on ON symptoms. This pattern was also seen when examining the EHQ subscales of knowledge about healthy eating, χ^2^(4) = 16.31, *p* = 0.003, Cramer’s V = 0.35, and positive feelings about healthy eating, χ^2^(4) = 10.12, *p* = 0.038, Cramer’s V = 0.28. More specifically, those who scored low on EHQ-Knowledge were more likely to report feeling “somewhat less” (*z* = 2.93, *p* = 0.003) or “a lot less” (*z* = 2.39, *p* = 0.017) pressure to lose weight since the start of the pandemic than the high EHQ-Knowledge group. Those low on EHQ-Feelings reported “a lot less” perceived pressure to lose weight than those high on EHQ-Feelings (*z* = 2.52, *p* = 0.012).

The two ON symptoms groups also significantly differed in their perceived pressure to diet, χ^2^(4) = 34.02, *p* < 0.001, Cramer’s V = 0.52, such that those low on ON symptoms were more likely to report “about the same” (*z* = 3.63, *p* < 0.001) or “somewhat less” (*z* = 2.42, *p* = 0.016) pressure to diet than before lockdown began as compared to the high ON symptom group. In other words, women low on ON were disproportionately likely to say that they felt less pressure to diet during lockdown. This difference was found to be marginally significant when examining the EHQ subscale of feelings about healthy eating, χ^2^(4) = 9.48, *p* = 0.050, Cramer’s V = 0.27. Individuals who scored low on EHQ-Feelings were more likely to report “somewhat less” perceived pressure to diet during the pandemic than those who scored high on EHQ-Feelings (*z* = 1.94, *p* = 0.052).

Beyond perceived pressure and attitudes about weight loss, there was also a significant difference between the two ON symptom groups in regard to whether they chose to start a diet during lockdown or not, *F* (1,141) = 4.31, *p* = 0.040. On average, those high on ON symptoms (*M* = 1.61, *SD* = 0.49) were more likely to endorse having started a diet during the lockdown period than those in the low ON symptom group (*M* = 1.77, *SD* = 0.43). When examining subscales of the ON measure there was a trend toward significant differences in laxative use on the EHQ-Feelings subscale, χ^2^(1) = 4.24, *p* = 0.039, Cramer’s V = 0.28. More specifically, those who scored low on EHQ-Feelings were more likely to report “about the same to a lot less” laxative use during the lockdown (*z* = 2.06, *p* = 0.039) as compared to the high EHQ-Feelings group, while those who scored high on positive feelings toward healthy eating were more likely to report “somewhat to a lot more” laxative use (*z* = −2.06, *p* = 0.039) since the start of the pandemic. See Table 2 for statistical test results.

**TABLE 2:** Items related to dieting attitudes and behaviors (chi-squared tests).

Items			χ ^2^	*df*	*z*	*p*	Cramer’s V
**Pressure to lose weight**							
	Total		27.51	4	–	<0.001	0.46
		A lot less	–	–	3.64	<0.001	–
		Somewhat less	–	–	0.31	0.757	–
		About the same	–	–	2.32	0.020	–
		Somewhat more	–	–	−0.65	0.516	–
		A lot more	–	–	−4.05	<0.001	–
	EHQ-Knowledge		16.31	4	–	0.003	0.35
		A lot less	–	–	2.39	0.017	–
		Somewhat less	–	–	2.93	0.003	–
		About the same	–	–	−0.46	0.646	–
		Somewhat more	–	–	−1.83	0.067	–
		A lot more	–	–	−0.85	0.395	–
	EHQ-Feelings		10.12	4	–	0.038	0.28
		A lot less	–	–	2.52	0.012	–
		Somewhat less	–	–	1.53	0.126	–
		About the same	–	–	−1.49	0.136	–
		Somewhat more	–	–	−0.81	0.418	–
		A lot more	–	–	−0.23	0.818	–
**Pressure to diet**							
	Total		34.02	4	–	<0.001	0.52
		A lot less	–	–	1.56	0.119	–
		Somewhat less	–	–	2.42	0.016	–
		About the same	–	–	3.63	<0.001	–
		Somewhat more	–	–	−1.19	0.230	–
		A lot more	–	–	−4.61	<0.001	–
	EHQ-Feelings		9.48	4	–	0.050	0.27
		A lot less	–	–	1.61	0.107	–
		Somewhat less	–	–	1.94	0.052	–
		About the same	–	–	−1.64	0.101	–
		Somewhat more	–	–	−1.48	0.139	–
		A lot more	–	–	0.14	0.889	–
**Laxative use**							
	EHQ-Feelings		4.24	1	–	0.039	0.28
		A lot less, somewhat less, or about the same	–	–	2.06	0.039	–
		Somewhat more or a lot more	–	–	−2.06	0.039	–

### Social Media Use and Influence

There was a significant difference between the two ON symptom groups in regard to perceived pressure from social media to lose weight, χ^2^(2) = 16.96, *p* < 0.001, Cramer’s V = 0.39, whereby the low ON symptom group was more likely to report “somewhat to a lot less” pressure (*z* = 3.27, *p* < 0.001) than the high ON symptoms group. On the other hand, the high ON symptom group was more disproportionately more likely to report “somewhat to a lot more” pressure to lose weight coming from social media (*z* = −3.65, *p* < 0.001) than their low ON symptom counterparts. In addition, there was a significant difference on this item for the subscale of feelings toward healthy eating, χ^2^(2) = 8.35, *p* = 0.015, Cramer’s V = 0.27. Those low on EHQ-Feelings reported “somewhat to a lot less” pressure (*z* = 2.31, *p* = 0.021) whereas those high on EHQ-Feelings reported experiencing “about the same” amount of pressure as before the lockdown began (*z* = −2.29, *p* = 0.022).

Both high and low ON symptom groups reported significant changes on perceived pressure from social media to exercise since the start of the pandemic, χ^2^(2) = 15.33, *p* < 0.001, Cramer’s V = 0.36, but in opposite directions. The low ON symptom group were more likely than the high ON symptoms group to report “somewhat to a lot less” pressure from social media to exercise (*z* = 2.59, *p* = 0.009). On the other hand, the high ON symptom group were more likely to report either “about the same” (*z* = 2.34, *p* = 0.019) or “somewhat to a lot more” (*z* = −3.80, *p* < 0.001) pressure from social media to exercise since the start of the pandemic than the low ON symptoms group. This reveals that whereas women low on ON symptoms were disproportionately more likely to say that they felt less pressure to exercise, those high on ON symptoms were disproportionately more likely to say that they felt more pressure to exercise due to social media.

This pattern was also found when comparing low vs. high scorers on the EHQ-Feelings subscale, χ^2^(2) = 6.29, *p* = 0.043, Cramer’s V = 0.23, such that individuals who scored low on that subscale were more likely to report “somewhat to a lot less” pressure to exercise from social media compared to those who score high on that subscale (*z* = 2.50, *p* = 0.012). See Table 3 for statistical test results.

**TABLE 3:** Items related to social media use (chi-squared tests).

Items			χ^2^	*df*	*z*	*p*	Cramer’s V
**Perceived pressure from social media to lose weight**							
	Total		16.96	2	–	<0.001	0.39
		A lot less or somewhat less	–	–	3.27	<0.001	–
		About the same	–	–	1.52	0.129	–
		Somewhat more or a lot more	–	–	−3.65	<0.001	–
	EHQ-Feelings		8.35	2	–	0.015	0.27
		A lot less or somewhat less	–	–	2.31	0.021	–
		About the same	–	–	−2.29	0.022	–
		Somewhat more or a lot more	–	–	0.59	0.555	–
**Perceived pressure from social media to exercise**							
	Total		15.33	2	–	<0.001	0.36
		A lot less or somewhat less	–	–	2.59	0.009	–
		About the same	–	–	2.34	0.019	–
		Somewhat more or a lot more	–	–	−3.80	<0.001	–
	EHQ-Feelings		6.29	2	–	0.043	0.23
		A lot less or somewhat less	–	–	2.50	0.012	–
		About the same	–	–	−0.70	0.484	–
		Somewhat more or a lot more	–	–	−0.98	0.327	–

Notably, there were marginally significant differences in the hours spent scrolling through diet accounts on social media (i.e., accounts whose posts promote clean eating or health-related diets) each day, *F* (1,141) = 3.82, *p* = 0.053. Those high on ON symptoms reported slightly more time on social media (*M* = 52.2 min, *SD* = 49.2 min) than those low on ON symptoms (*M* = 36.6 min, *SD* = 28.8 min). When examining the subscales, women who scored high on EHQ-Problems described seeing more content on social media about losing weight during the lockdown (*M* = 1.73, *SD* = 0.45) than those who scored low on that subscale (*M* = 1.57, *SD* = 0.50), *F* (1,141) = 3.74, *p* = 0.055. However, the high and low scoring groups on EHQ-Problems were equally likely to report more time spent on Facebook, χ^2^(4) = 2.18, *p* = 0.703, Instagram, χ^2^(2) = 2.44, *p* = 0.295, and TikTok, χ^2^(1) = 0.60, *p* = 0.437.

## Discussion

The COVID-19 pandemic has had adverse effects both on mental health and on eating and related constructs for many individuals across the globe. In addition to expanding the literature on the deleterious effects of pandemic lockdowns on well-being (see Novotný et al., 2020), the current study sought to expand our understanding of the psychology of the proposed eating disorder orthorexia nervosa (ON). Individuals with an eating disorder have been previously thought to be especially vulnerable to an exacerbation of symptoms during this pandemic. To our knowledge, this study is the first to examine the effects of a pandemic lockdown on individuals with symptoms of ON—a pathological preoccupation with clean or healthy eating. This study examined reported changes to adults’ eating habits, exercise, and social media use during a COVID-19 lockdown period in Canada, and to compare women with and without symptoms of ON.

We recruited women across the adult lifespan; women in the high ON symptom group were generally the same age as those in the low ON symptom group, with the average age in both groups being 25 years old. Interestingly, we found that the high ON symptom group reported being heavier than the low ON symptom group; the high ON group average BMI fell in the “overweight” range and the low ON group’s average fell in the “healthy” range according to World Health Organization BMI categories (WHO, 1995). There is scarce and mixed literature on the weight status of individuals with ON (McComb and Mills, 2019). The current findings suggest that despite ON sharing features with anorexia nervosa (Gramaglia, 2017), women with ON may, if anything, tend to be heavier relative to women without ON. More research is needed to verify whether this is a reliable finding. However, the current findings suggest that a pathological obsession with clean or healthy eating does not necessarily manifest as being underweight.

It was hypothesized that women high on ON symptoms would report increased disordered eating habits, worsened body image, and would report being more negatively affected by social media as compared to those low on ON symptoms and since the start of lockdown. The findings supported our hypotheses in the following inter-related areas of inquiry: (1) eating behavior and thoughts about food, (2) body image and weight, and (3) pressure from social media. The findings for each area of inquiry are discussed below.

### Eating and Thoughts About Food

When asked about changes they had experienced since the lockdown period began, women high on ON symptoms, but not women low on ON symptoms, were more likely to report undesirable changes to their eating behavior, including eating a lot more food than usual and thinking about food more often than usual since lockdown began. These findings suggest that women who are extremely preoccupied with eating clean or healthy food were more negatively affected by the lockdown in terms of eating and thoughts about food. Furthermore, the findings suggest that their eating behavior during lockdown was in the *opposite* direction of what individuals with ON are motivated to do, which includes eliminating a great deal of foods from their diet so as to be healthier. Increased food consumption is not in line with the goals of ON. One of the more reliable findings from past studies of changes to eating behavior during the COVID-19 pandemic is the reporting of increased food consumption (i.e., snacking among the general population, binging among individuals with eating disorders). In the current study, that result was replicated; however, increased food consumption and thinking more about food was reported only by the women high on ON symptoms and not by women without symptoms of ON.

### Body Image and Dieting

Another consequence of the lockdown on women high on ON symptoms, but not those low on ON symptoms, was that they reported that they had gained a significant amount of weight during lockdown, felt increased pressure to lose weight, and were likely to have started a diet during lockdown. Together, these findings are evidence that the lockdown worsened various aspects of body image among women with ON tendencies. As is true among individuals with a traditional eating disorder, who have been shown to be vulnerable to worsened symptoms during the pandemic (Brown et al., 2021), the current study found that the lockdown triggered negative body image and increased dieting behavior in women high on ON symptoms. Women with low ON symptoms, on the other hand, were most likely to state that their weight had stayed the same during lockdown. Although we cannot test these associations causally with the available data, the findings suggest that the underlying psychology of ON is similar to other eating disorders and that body dissatisfaction is triggered when individuals feel as if they have eaten more than usual. When obsessive control over one’s eating fails, negative body image and urges to diet tend to increase. In the case of ON, disruptions to one’s ability to carefully monitor and control the “healthiness” of one’s diet is clearly distressing to women with ON in terms of body image. This is consistent with other research suggesting individuals with ON experience value thinness and when they feel as if they have overeaten, they experience elevated levels of body dissatisfaction (see McComb and Mills, 2019).

What is interesting to consider, and unclear from the available data, is whether eating and/or weight changes were truly or objectively different between the two ON groups or whether the high ON symptom group was more *bothered* by disruptions to their eating due to lockdown. It could be that women who are not preoccupied with their diet are able to show more flexibility and to better tolerate the dietary and lifestyle changes they faced during lockdown. As such, those same women are less bothered by and/or less likely to report undesirable consequences of the lockdown. More research is needed, preferably with prospective designs and data that are not only self-report (e.g., behavioral, other-reports, collection of objective weight data).

### Pressure From Social Media

As reviewed earlier, there was a surge in social media use during the COVID-19 pandemic as people spent more time as home and were more socially isolated. This is the first study to our knowledge to examine perceived pressure from social media during the COVID-19 pandemic; this is a novel contribution of the current study to the literature on the effects of the COVID-19 pandemic on adult women. The current study found support for the hypothesis that social media pressure worsened during the lockdown, but only for women high on ON symptoms. Specifically, women high on ON symptoms felt more pressure from social media to lose weight during lockdown. Conversely, women low on ON symptoms actually felt *less* pressure from social media to lose weight during lockdown. This could be because they were paying more attention to COVID-19-related news during that time and paying less attention to appearance-related content. Women high on ON tendencies may have a hard time ignoring weight-based content on social media or even have sought it out more than usual during lockdown, as a result of the body dissatisfaction and perceived changes to their eating discussed above.

Taking a detailed look at the different facets of ON as measured by the EHQ, we examined associations between individual subscales and perceived pressure from social media. We had no formal hypotheses but were curious as to whether different subscales on the EHQ measure of ON might reveal different patterns of effects. Women who endorsed *problems* caused by their healthy eating reported seeing more weight loss content on their social media during the pandemic than those who reported few ON symptoms. Women who endorsed *knowledge* of healthy behaviors were more likely to report feeling either somewhat more or a lot more pressure to lose weight than those who reported fewer ON symptoms. And women who endorsed *feeling positive* about their healthy eating tended to report a lot more pressure to lose weight, both somewhat more or a lot more pressure to lose weight or to exercise from social media specifically, and trended toward more laxative use during the lockdown period of study, compared to those who scored lower on items endorsing positive feelings about healthy eating. These results need to be replicated with a larger sample. However, they suggest that feeling positive about extreme healthy eating may be an especially problematic symptom of ON. In the context of disordered eating, laxative use is a very problematic behavior aimed at weight loss and/or reducing body dissatisfaction due to stomach bloating. This observed trend of increased laxative use during lockdown among women who feel good about their ON symptoms is cause for concern. Taken together, the responses of this group of women who feel good about their ON symptoms and the sense of control those symptoms give them suggests a lack of insight into when healthy eating crosses the line and becomes pathological. For these individuals, extreme healthy eating could be an emotion regulation strategy that is ego syntonic. Emotion regulation is implicated in other forms of disordered eating (e.g., Shakory et al., 2015) and further research into ON as an emotion regulation strategy is warranted. In turn, when attempts at healthy eating fail, they may turn to other compensatory behaviors, such as laxatives. Taken together, these findings provide evidence of the influence of social media on some women’s perceived pressure to lose weight during the pandemic. Because social media use has increased during the pandemic, this form of media may be more important than ever as an influence on adult women’s body image. Future research should investigate the link between social media and ON since this may be an especially powerful way by which disordered attitudes about so-called “healthy” eating are learned.

### Possible Explanations

Although beyond the scope of the current study, there are numerous reasons why lockdowns during the COVID-19 pandemic might worsen any type of disordered eating, including ON. Fitness centers and gym closures may increase concern that one will gain weight. Food restriction can result from contagion fears around grocery shopping. Social isolation and less control over time spent with family and roommates may cause significant stress that can manifest in many of the results we found here: overeating, dieting, body dissatisfaction, and perceived pressure to lose weight. Importantly, loneliness and sadness may increase engagement in harmful behaviors such as overeating and dieting as a way to cope, as has been documented with respect to substance use (Wardell et al., 2020). Other research has shown that loneliness in particular predicts surges in mental distress during lockdowns among the general population (Novotný et al., 2020) and should be a construct of interest in future research on disordered eating. We speculate that individuals with eating disorders, including ON, might struggle more with their eating during times of social isolation as they retreat more into their disordered eating as a way of coping with uncertainty and stress. Eating can become a major life focus when there is little else to do outside of the home. Stress-induced overeating or abandonment of previously restrictive eating may have deleterious effects on body image, which can fuel a vicious cycle of restriction and overeating. The current study design cannot tease apart the myriad factors mediating the changes that women reported, but can inform future research.

### Strengths and Limitations

The current findings contribute to the growing body of literature on the nature of ON. Much of the previous research has been marred by invalid measurement of ON and has been inconclusive regarding the motivation underlying ON tendencies (McComb and Mills, 2019). A strength of the current study is the use of a psychometrically sound measure of ON, the EHQ. Importantly, this study highlights the importance of body image, a drive for thinness, and a particular vulnerability to perceived social pressures to achieve a thin body that characterizes individuals with ON tendencies. While most of the existing research on ON treats weight loss as a *consequence* of restricting one’s diet in the pursuit of clean or health eating, this study suggests that the stress of the current pandemic has provoked even more of a reoccupation with weight loss, dieting, and exercise among these individuals.

One limitation of this study is the use of self-report data for ON symptoms. Although we utilized a well-supported self-report measure of ON (McComb and Mills, 2019), there may be nuances and characteristics better assessed through a psychodiagnostic interview with a trained clinician. Moreover, because the ON measure was completed in after the start of the global pandemic, this study cannot isolate whether these symptoms are longstanding or emerged during the pandemic. However, given the unique circumstance of this social event and, thus, the inability to collect prospective data, this form of data collection allows for preliminary insight into the experiences of people with ON tendencies. Another limitation is the modest sample size. Although the findings were statistically significant, a larger sample and replication would increase confidence in these findings. We did not have a large enough sample (or enough statistical power) to perform a tertile split based on EHQ score, which would offer a more accurate picture of the differences between truly high and low ON tendencies than a median split. This should be considered in future studies. Finally, another limitation of the study is that it included only women. Dieting, drive for thinness, and disordered eating are highly gendered constructs and more common among women than men. Because of a finite amount of time available to collect data during the lockdown period, we narrowed recruitment to just female participants. The current findings cannot be generalized to men with and without ON tendencies, which is an area for future research.

### Clinical Implications

During the SARS epidemic in 2003, studies reported elevated levels of anxiety and depression that persisted 3 years later (Liu et al., 2012), with those under quarantine showing a dramatic increase in post-traumatic stress symptoms (Hawryluck et al., 2004; Liu et al., 2012). COVID-19 has been an even more significant disruptor. The current findings suggest that, like individuals with the traditional eating disorder diagnoses, women who are high on ON tendencies should be monitored carefully in the coming years and may need clinical intervention. EDs are an especially stubborn form of psychopathology, showing less evidence of natural recovery in young adults than anxiety or depression symptoms (Mills et al., 2012), and ON might show a similar trend. The current findings underscore the seriousness of ON and a need for evidence-based primary prevention programs, such as media literacy, and other evidence-based strategies for reducing the impact of an EDs (see Stice et al., 2013), and a non-dieting approach, which might discourage the development of ON (Varga et al., 2014). Educating at-risk youth and adults around “healthy” media messaging, especially on social media, is paramount. Health and thinness are often confounded across all forms of media. Much of “health-related” messaging is actually anti-fat messaging that stigmatizes people in larger bodies (e.g., Puhl and Heuer, 2010). Stress management, mindfulness practice, and emotion regulation skills training may all be useful in building resilience and can prevent worsening of disordered eating symptoms, including ON, in the face of unforeseen stressors. Individual with an eating disorder rated virtual social interactions with friends, moderate physical activity, and pleasurable activities as the most helpful strategies to aid with their disorder during the pandemic (Schlegl et al., 2020).

## Conclusion

Women who endorsed high levels of ON symptoms experienced an exacerbation of disordered eating thoughts and behaviors during a period of lockdown 3 months into the COVID-19 pandemic. Women high on ON symptoms reported eating a lot more food and thinking about food more often, being more likely to have started a diet, feeling greater pressure to diet and to lose weight, experiencing greater weight gain, and perceiving more pressure from social media specifically to lose weight and to exercise, compared to women without ON. Social media may be a contributing factor to worsened body image during the pandemic.

## Data Availability Statement

The raw data supporting the conclusions of this article will be made available by the authors, without undue reservation.

## Ethics Statement

The studies involving human participants were reviewed and approved by York University Human Participants Review Committee. The patients/participants provided their written informed consent to participate in this study.

## Author Contributions

KG, JM, and SM contributed to conception and design of the study. KG and SM organized the database. KG performed the statistical analysis. JM and KG wrote the first draft of the manuscript. All authors contributed to manuscript revision, read, and approved the submitted version.

## Conflict of Interest

The authors declare that the research was conducted in the absence of any commercial or financial relationships that could be construed as a potential conflict of interest.

## Publisher’s Note

All claims expressed in this article are solely those of the authors and do not necessarily represent those of their affiliated organizations, or those of the publisher, the editors and the reviewers. Any product that may be evaluated in this article, or claim that may be made by its manufacturer, is not guaranteed or endorsed by the publisher.
